# Kisspeptin-10 Mitigates α-Synuclein-Mediated Mitochondrial Apoptosis in SH-SY5Y-Derived Neurons via a Kisspeptin Receptor-Independent Manner

**DOI:** 10.3390/ijms24076056

**Published:** 2023-03-23

**Authors:** Christopher Simon, Tomoko Soga, Ishwar Parhar

**Affiliations:** Jeffrey Cheah School of Medicine and Health Sciences, Monash University Malaysia, Bandar Sunway 47500, Selangor, Malaysia; christopher.simon@monash.edu (C.S.); tomoko.soga@monash.edu (T.S.)

**Keywords:** choline acetyltransferase, cholinergic neurons, dementia with Lewy bodies, E46K mutant, GPR54, intrinsic apoptosis, kisspeptin-234, mitochondrial depolarization, neuroprotection

## Abstract

The hypothalamic neurohormone kisspeptin-10 (KP-10) was inherently implicated in cholinergic pathologies when aberrant fluctuations of expression patterns and receptor densities were discerned in neurodegenerative micromilieus. That said, despite variable degrees of functional redundancy, KP-10, which is biologically governed by its cognate G-protein-coupled receptor, GPR54, attenuated the progressive demise of α-synuclein (α-syn)-rich cholinergic-like neurons. Under explicitly modeled environments, in silico algorithms further rationalized the surface complementarities between KP-10 and α-syn when KP-10 was unambiguously accommodated in the C-terminal binding pockets of α-syn. Indeed, the neuroprotective relevance of KP-10’s binding mechanisms can be insinuated in the amelioration of α-syn-mediated neurotoxicity; yet it is obscure whether these extenuative circumstances are contingent upon prior GPR54 activation. Herein, choline acetyltransferase (ChAT)-positive SH-SY5Y neurons were engineered ad hoc to transiently overexpress human wild-type or E46K mutant α-syn while the mitigation of α-syn-induced neuronal death was ascertained via flow cytometric and immunocytochemical quantification. Recapitulating the specificity observed on cell viability, exogenously administered KP-10 (0.1 µM) substantially suppressed wild-type and E46K mutant α-syn-mediated apoptosis and mitochondrial depolarization in cholinergic differentiated neurons. In particular, co-administrations with a GPR54 antagonist, kisspeptin-234 (KP-234), failed to abrogate the robust neuroprotection elicited by KP-10, thereby signifying a GPR54 dispensable mechanism of action. Consistent with these observations, KP-10 treatment further diminished α-syn and ChAT immunoreactivity in neurons overexpressing wild-type and E46K mutant α-syn. Overall, these findings lend additional credence to the previous notion that KP-10’s binding zone may harness efficacious moieties of neuroprotective intent.

## 1. Introduction

Pervasive dissemination of α-synuclein (α-syn)-laden inclusions in the immediate vicinity of cholinergic neurons exemplifies the defining neuropathological phenotype of dementia with Lewy Bodies (DLB) [1,2]. The sporadic manifestations of these anomalous deposits, albeit paralleled by aberrant increments in wild-type α-syn expression [3], have been causally linked to familial pedigrees bearing the E46K mutation [4]. Indeed, irrespective of whether these lesions are themselves pathogenic, their omnipresence reflects the functional compromise of α-syn, a presynaptically-localized 140 amino acid protein [5]. While the biological purpose of α-syn remains indeterminate, at supraphysiological levels, α-syn may be extruded in a stochastic manner from an initiating subpopulation of selectively vulnerable neurons [6]. The consequence is a myriad of pathogenically variant deposits, each of which entails the premature accretion of α-syn in characteristic patterns and locations [7,8,9]. Reminiscent of prion-like transmissibility [10], the α-syn aggregates are sequestered into juxtanuclear inclusions or deleterious assemblies and may underlie mitochondrial depolarization, which is known to predate the apoptotic demise of neurons [11,12]. In more obscure circumstances, the coalescence of α-syn into neurotoxic lesions is interspersed with choline acetyltransferase (ChAT)-positive neurons [13]; the abundance of which appears to reflect a precipitous decline in premorbid cognitive functioning [14,15]. It is, therefore, conceivable that an effective α-syn-centric intervention must be initiated prophylactically before the self-perpetuating cascade progresses beyond initial predilection sites. In this regard, the unconventional alterations of a hypothalamic neuropeptide, termed kisspeptins, in dementia-prone limbic regions [16,17] underpinned an unprecedented dimension to the advent of neurotherapeutics in α-syn-driven lesions. While the pathophysiological instigation of this seminal occurrence was well beyond the strict confines of the gonadotrophic axis [17], the corollary that it may be neuroprotective against degenerative pathologies is likewise gaining traction. An irrefutable gatekeeper of pubertal function [18], kisspeptin-10 (KP-10), which is proteolytically liberated from a 145 amino acid precursor protein and biologically governed by its G-protein-coupled receptor, GPR54 [19], inhibited the noxious insults of extracellular amyloid β-proteins (Aβ) via an action that GPR54 antagonists could not impede [20]. Concomitantly, genetic ablation of the *KiSS-1* gene, which encodes kisspeptin, exacerbated Aβ-induced toxicity, while *KiSS-1*-overexpressing neurons established a cellular milieu that was impervious to Aβ toxicity [20,21]. Paralleling these observations, recent preliminary findings from our laboratory demonstrated that exogenously administered KP-10 was efficacious in attenuating the progressive demise of α-syn-rich cholinergic neurons in vitro [22]. Under explicitly modeled environments, KP-10 unambiguously accommodated in the C-terminal binding pockets of α-syn despite solvent-driven perturbations, thus reflecting the existence of a putative binding site [22]. Clearly, the functional relevance of KP-10 binding mechanisms can be insinuated in the amelioration of α-syn-mediated neurotoxicity; yet it is obscure whether KP-10’s mode of protection is contingent upon GPR54 activation. At its core, the binary engagements between KP-10 and GPR54 set in motion a signal-transducing phenomenon that employs the concerted liberation of G_q/11_-derived G subunits [23,24]. One of these, the Gα_q_ subunit, physically interdicts the obligatory phospholipase C (PLC)-catalyzed hydrolysis of phosphatidylinositol 4,5-bisphosphate (PIP_2_) into inositol 1,4,5-trisphosphate (IP_3_) and diacylglycerol (DAG) [18,25]. Consequently, the former incites an upsurge in cytosolic Ca^2+^, while the latter activates protein kinase C (PKC), which in turn phosphorylates mitogen-activated protein (MAP) kinases p38 and extracellular signal-regulated kinase (ERK) [26,27]. Given GPR54’s functional repertoire, the stringency with which this canonical Gα_q_-dependent pathway function is thus undeniably discriminative in their recognition of substrates [19]. Nevertheless, despite its physiological relevance, a cell-dependent dichotomy in signaling function was seemingly imminent when GPR54-mediated potentiation of β-arrestin 2 initiated the ERK1/2 signal transduction network following KP-10 stimulation [28,29]. Empowered by this intricate but tiered signaling cascade, the subsequent downstream activation of the phosphatidylinositol 3-kinase (PI3K)/AKT/glycogen synthase kinase-3β (GSK3β) pathway was aberrantly ameliorative against mitochondrial superoxide and apoptotic insults [30]. Hence, having established a human cholinergic cellular model that overexpresses wild-type or E46K mutant α-syn previously [22], the present investigation attempts to delineate the mechanistic underpinnings of KP-10’s mode of neuroprotection by means of a GPR54 antagonist.

## 2. Results

### 2.1. KP-10 Attenuates α-Syn-Mediated Apoptotic Death in Cholinergic-like Neurons via a GPR54-Independent Manner

**Figure 1:**
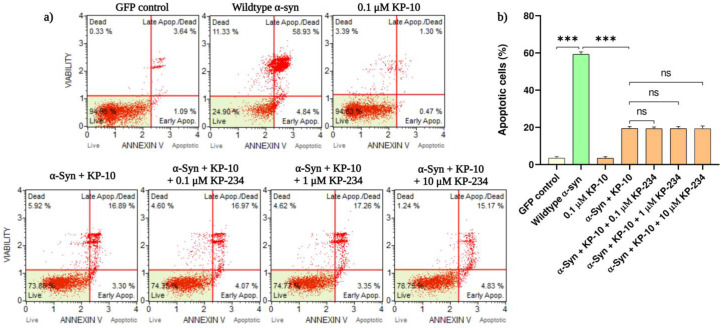

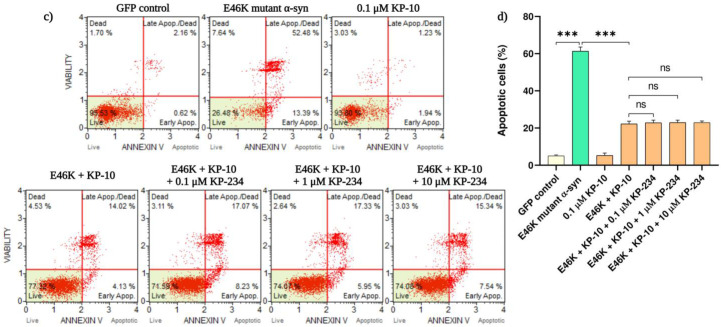
The effect of kisspeptin-10 (KP-10) on human wild-type or E46K mutant α-synuclein (α-syn)-induced apoptosis in cholinergic-like neurons. Differentiated neurons overexpressing human wild-type or E46K mutant α-syn were exogenously administered with KP-10 (0.1 µM) either alone or in combination with KP-234 (0.1 to 10 µM). The induction of apoptotic death was measured using the Muse automated cell analyzer. (**a**,**c**) Representative plots of experiments executed for annexin-V detection showing non-apoptotic live (**lower left**: 7-AAD negative, apoptosis negative), non-apoptotic dead (**upper left**: 7-AAD positive, apoptosis negative), apoptotic live (**lower right**: 7-AAD negative, apoptosis positive), and apoptotic dead (**upper right**: 7-AAD positive, apoptosis positive) cells. The quantification of total apoptotic cells presented in (**b**,**d**) is representative of mean values ± SEM from three independent biological replicates performed in triplicates. While the externalization of plasma membrane phosphatidylserine by both human wild-type (**a**,**b**) and E46K mutant (**c**,**d**) α-syn was considerably inhibited by KP-10 (*** *p* < 0.001), co-administrations with KP-234 had no discernible impact on this ameliorative effect. Statistical significance was defined as *p*-value less than 0.001 (*** *p* < 0.001).

Colorimetric quantification of viable cells from our recently established cholinergic model demonstrated that profound upregulation of α-syn mRNA expression upon transfection is metabolically detrimental [22]. On this basis, the apoptotic consequences of transiently transfected α-syn constructs on cholinergic differentiated neurons were consecutively analyzed by means of an annexin-V-affinity assay. Herein, we report that significant increases in apoptotic demise were discerned amongst neurons overexpressing human wild-type (59.3 ± 1.2%, *** *p* < 0.001) and E46K mutant α-syn (61.4 ± 2.2%, *** *p* < 0.001) compared with the GFP-positive neurons. In an attempt to further delineate the neuroprotective relevance of KP-10/GPR54 signaling in α-syn-induced apoptosis, transfected neurons were exogenously treated with KP-10 (0.1 µM) either alone or in concert with KP-234 (0.1 to 10 µM). Intriguingly, while the apoptosis-dependent externalization of phosphatidylserine by both human wild-type and mutant α-syn was substantially suppressed by KP-10 (*** *p* < 0.001), co-administrations with the GPR54 antagonist KP-234 had a negligible influence on this ameliorative effect (Figure 1a–d).

### 2.2. KP-10 Rescues α-Syn-Induced Mitochondrial Depolarization in Cholinergic-like Neurons through a GPR54 Dispensable Mechanism

**Figure 2:**
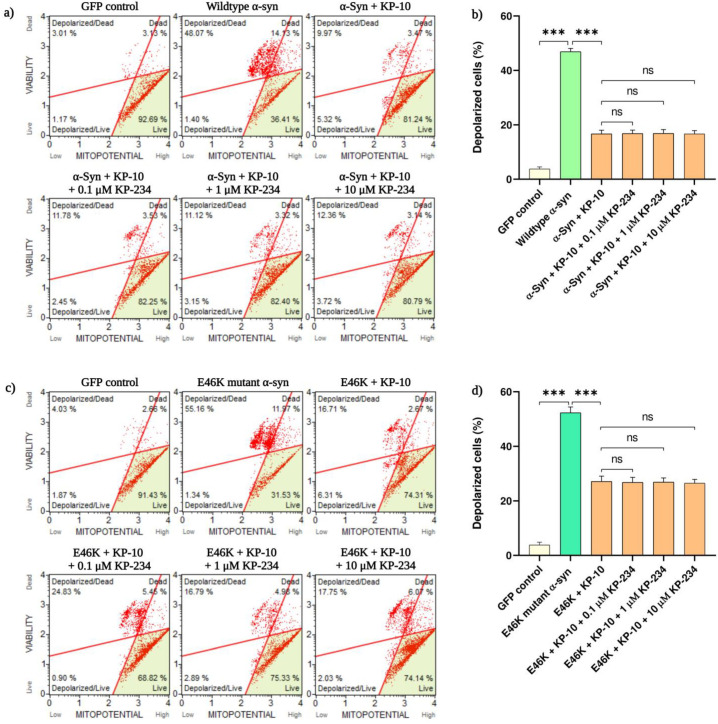
The effect of kisspeptin-10 (KP-10) on human wild-type or E46K mutant α-synuclein (α-syn)-mediated mitochondrial depolarization in cholinergic-like neurons. Differentiated neurons overexpressing human wild-type or E46K mutant α-syn were exogenously subjected to KP-10 (0.1 µM) either alone or in concert with KP-234 (0.1 to 10 µM). The induction of mitochondrial membrane depolarization was measured using the Muse automated cell analyzer. (**a**,**c**) Representative plots of experiments executed for the detection of mitochondrial depolarization showing depolarized live (**lower left**: 7-AAD negative, mitopotential negative), depolarized dead (**upper left**: 7-AAD positive, mitopotential negative), non-depolarized live (**lower right**: 7-AAD negative, mitopotential positive), and non-depolarized dead (**upper right**: 7-AAD positive, mitopotential positive) cells. The quantification of total depolarized cells presented in (**b**,**d**) is representative of mean values ± SEM from three independent biological replicates performed in triplicates. While the α-syn-mediated dissipation of mitochondrial membrane potential by both human wild-type (**a**,**b**) and E46K mutant (**c**,**d**) α-syn was substantially reverted by KP-10 (*** *p* < 0.001), co-administrations with KP-234 had a non-existent influence on this alleviative effect. Statistical significance was defined as *p*-value less than 0.001 (*** *p* < 0.001).

Working on the premise that α-syn-mediated lethality may, in part, necessitate the instigation of the intrinsic apoptotic cascade, mitochondrial integrity of α-syn-transfected neurons was subsequently probed by tracking the intramitochondrial sequestration of a membrane-permeant dye. Accordingly, flow cytometric analysis revealed a pronounced upsurge in mitochondrial depolarizations of neurons overexpressing human wild-type (46.9 ± 1.2%, *** *p* < 0.001) and E46K mutant α-syn (52.3 ± 2.1%, *** *p* < 0.001) compared with the GFP-labeled neurons. This α-syn-induced dissipation of membrane potential, albeit significant, was considerably reverted by the salutary effects of KP-10 (*** *p* < 0.001). Consequently, co-administrations with KP-234, however, had no significant impact on this prophylactic outcome (Figure 2a–d).

### 2.3. KP-10 Diminishes α-Syn and ChAT Immunofluorescence Intensity in Cholinergic-like Neurons Overexpressing Human Wild-Type or E46K Mutant α-Syn

**Figure 3:**
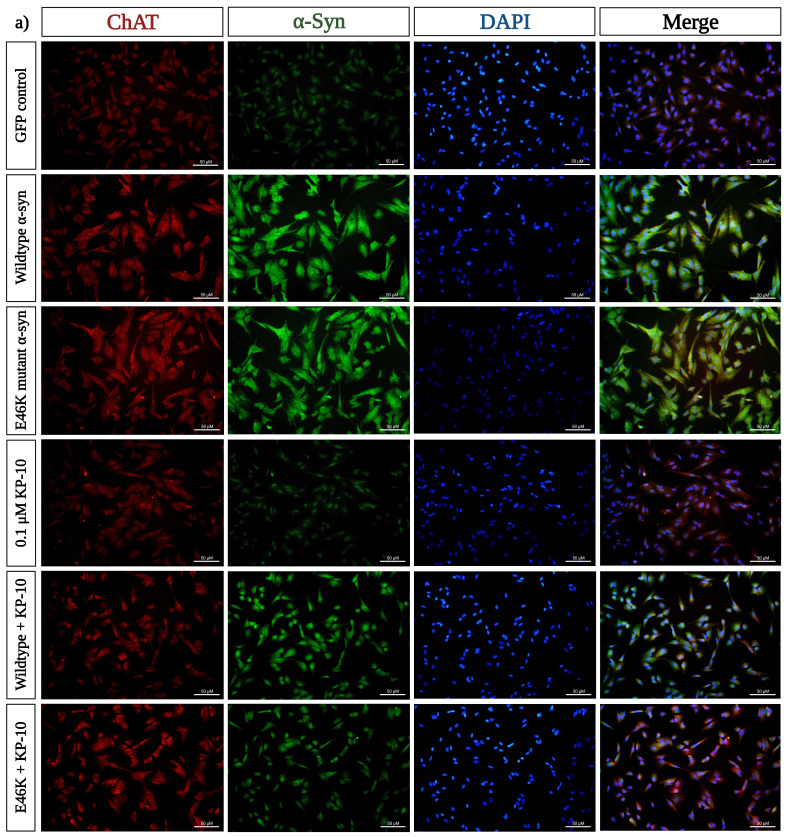

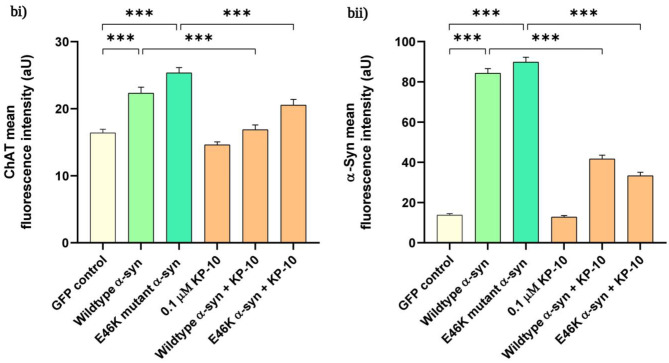
The effect of kisspeptin-10 (KP-10) on α-synuclein (α-syn) and choline acetyltransferase (ChAT) immunoreactivity in neurons overexpressing human wild-type or E46K mutant α-syn. (**a**) Differentiated neurons overexpressing human wild-type or E46K mutant α-syn were double-immunostained with anti-α-syn (green) and anti-ChAT (red) antibodies, co-stained with Dapi (blue), and representative images were subsequently taken before and after treatment with KP-10. Scale bars, 50 µm. In this context, 60 neurons were analyzed per experiment. The staining intensity and quantification of α-syn and ChAT expression levels presented in (**b**) are representative of mean values ± SEM from three independent biological experiments. Quantitative analysis of α-syn and ChAT immunoreactions revealed significant increases of fluorescence intensity in both human wild-type (*** *p* < 0.001) and E46K mutant α-syn (*** *p* < 0.001) expressing neurons compared with the GFP-labeled neurons. This α-syn-induced upsurge in immunostaining intensity was considerably diminished by the neuroprotective effects of KP-10 (*** *p* < 0.001). Statistical significance was defined as *p*-value less than 0.001 (*** *p* < 0.001).

Discordance in the mechanisms governing ChAT as an enzymatic entity has been shown to underlie mitochondrial apoptotic susceptibility in α-syn-rich cholinergic-like neurons [14,31,32,33]. Thus, with this rationale in mind, we performed fluorescence intensity quantification on differentiated transfected neurons immunostained for ChAT and α-syn, respectively. Here, we show that the immunofluorescence intensity of α-syn and ChAT was significantly augmented in differentiated neurons overexpressing human wild-type (*** *p* < 0.001) or E46K mutant α-syn (*** *p* < 0.001) compared with the GFP-positive neurons. Following exogenous KP-10 treatment, marked reductions in α-syn and ChAT immunoreaction intensity were simultaneously observed for both human wild-type (*** *p* < 0.001) and E46K mutant α-syn (*** *p* < 0.001) expressing neurons (Figure 3a,b).

## 3. Discussion

**Figure 4:**
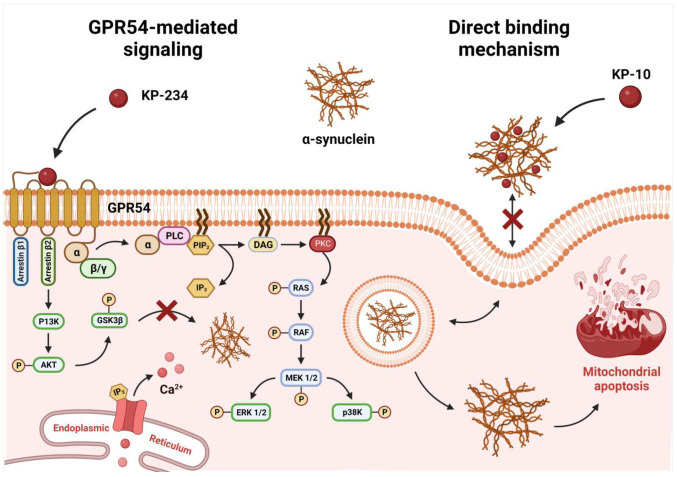
Hypothetical model depicting KP-10′s mode of protection in ChAT-positive neurons. A multi-hit hypothesis was proposed for KP-10′s alleviation of human wild-type and E46K mutant α-syn-induced neuronal death in ChAT-positive neurons, suggesting a direct-binding extracellular mechanism that directly or indirectly modulates ChAT expression levels. Supporting this notion, the pharmacological blockade of GPR54 with KP-234 had no influence on KP-10′s salutary effects.

Aberrant expressions of human wild-type and E46K mutant α-syn across cholinergic phenotypes have been causally linked to pathogenically noxious inclusions in the perturbed DLB microenvironment [34,35]. The widespread dissemination of these deleterious deposits, irrespective of whether it emanates intracellularly or extracellularly, may sensitize compromised neurons to spontaneous mitochondrial depolarizations, thereby rendering them more predisposed to apoptogenic consequences [11,36]. Since prophylactically intervening this commensurate loss of neurons has yet to result in disease-modifying therapeutics, there is an unmet requisite to counteract α-syn-centric extra- and intracellular pathogenic cascades. We have previously showcased that exogenous KP-10 was efficacious in diminishing the progressive demise of α-syn-rich ChAT-positive neurons with preferential docking affinities, hence reflecting a non-canonical extracellular binding interaction [22]. The recent revelation that GPR54 is endowed with a neuroprotective potential further buttressed the conjecture that KP-10 may engender a formerly unattainable level of pharmacologically-actionable specificity by additionally exploiting intracellular pro-survival signaling cascades [30]. Thus, with these premises in mind, cholinergic-like neurons were transiently engineered to overexpress human wild-type or E46K mutant α-syn, while KP-10′s mitigation of α-syn-induced neuronal death was probed by virtue of GPR54 antagonism. Herein, we report that neurons overexpressing human wild-type and E46K mutant α-syn were equally susceptible to apoptotic demise and temporally coincided with the dissipation of the mitochondrial transmembrane potential. Reiterating the specificity we observed preliminarily [22], exogenously administered KP-10 (0.1 µM) further repressed α-syn-mediated apoptosis and mitochondrial depolarization in cholinergic differentiated neurons. In particular, co-administrations with the GPR54 antagonist, kisspeptin-234, failed to nullify the robust neuroprotection elicited by KP-10, thereby signifying a GPR54 dispensable mechanism of action. Consistent with these findings, KP-10 treatment simultaneously diminished α-syn and ChAT expression levels in neurons overexpressing the human wild-type and E46K mutant α-syn. Indeed, if one were to extrapolate the results obtained from the above, it is conceivable that the preferential vulnerability of ChAT-positive neurons to mitochondrial apoptosis emanates from α-syn’s inherent propensity to modulate ChAT expression levels. The likelihood that this postulate has some merit has risen a notch or two [13,33], and it becomes reasonable to enquire as to how KP-10 restored ChAT expression to near-physiologic levels. As a seemingly reliable marker of anomalous cholinergic transmission [37,38], the reduction in ChAT expression is the most widely scrutinized facet of cholinergic pathology [39]. Nonetheless, spatial and temporal decreases in ChAT levels do not necessarily reflect cognitive deterioration considering that its theoretical capacity to synthesize acetylcholine is far in excess of the actual rate of acetylcholine biosynthesis [38,40]. Instead, paradoxical upregulation of ChAT expression has been ascertained in prodromal states, thus insinuating that such counterintuitive manifestations may compensate in unique chemoplastic fashions upon interactions with α-syn-enriched lesions [14]. As such, for neurotherapeutic initiatives to be efficacious, they must first harbor the latent ability to foster intrinsic and extrinsic pathways of neuronal integrity [41,42]. In this sense, we have identified KP-10 as a neuroprotectant that averts intrinsic apoptosis by modulating the externalization of plasma membrane phosphatidylserine and permeabilization of mitochondrial membranes. Mechanistically, the concerted action of GPR54 signaling kinases that operate either upstream or downstream of the aforementioned events is believed to account for whether neurons survive or succumb to α-syn’s noxious insults. The manner by which these downstream effectors are selectively governed, however, remains ill-defined since the pharmacological blockade of GPR54 had no discernible influence on KP-10′s salutary effects. At first consideration, the possibility of an inadequate receptor blockade cannot be entirely precluded, given that a certain degree of disparity in terms of antagonistic potency has been apparent under various experimental conditions [43,44,45,46]. Alternatively, the basis for such a phenomenon could also be indicative of a bona fide neuroprotective maneuver since these observations appear to resonate with our previously established in silico models, which subserved transient KP-10-α-syn complexations [22]. While the dynamical details of these explicitly modeled “adducts” provided more ambiguity on KP-10′s non-classical functional versatility, such dichotomies in modes of action were crucial in redirecting aggregation-predisposed α-syn into off-pathway non-toxic accretions [47]. Intriguingly, tertiary interactions with the C-terminal residues of α-syn were able to safeguard the C-terminus from bimolecular self-assembly and steer the expansion of a loop that is no longer primed for the generation of β-sheet-rich aggregates [48,49,50,51,52,53,54]. Though not exhaustive, there seems to be a basis for the apparent redundancy of GPR54 signaling in SH-SY5Y-derived neurons, for ideally, an insurmountable antagonist should considerably annul the intrinsic activity of an agonist [43,46]. Guided by this notion, the systematic substitution of amino acid residues along the core of KP-10 initially established a consensus sequence for antagonism that retained high-affinity receptor engagement but failed to elicit signaling in GPR54-expressing neurons [44]. To reconcile with the pleiotropic nature of its analog, KP-234 carries a substitution of Tyr^1^ with D-Ala and of Ser^5^ with Gly, with further enrichment by the replacement of Leu^8^ with D-Trp [44]. The fact that Tyr^1^ and Ser^5^ appear to constitute a binding pharmacophore that must be retained to effectuate KP-10-α-syn interactions [22] imparts credence to the notion that KP-234′s binding zone may not be able to harness functional moieties of protective relevance. With continued expansions in large-scale co-immunoprecipitations amidst GPR54-ablated neurons, established means of direct protein-protein interactions between KP-10 and α-syn will be essential for reconciling our initial theoretical predictions. Undeniably, the structural commonalities between KP-10 and α-syn made it tempting to propose that it is the extracellular interaction between these two proteins that drove the concerted attenuation of α-syn and ChAT expression levels. This hypothetical model, which is tentatively depicted in Figure 4, integrates the remodeling of toxic fibrils into non-toxic aggregates, yet whether the nature of this direct-binding interaction is confounded by the intricately woven proteostatic burden α-syn creates, warrants further clarification. While it may be of heuristic value to causally insinuate KP-10′s functionality in this regard, immunofluorescence analyses of α-syn clearance using autophagy-specific and aggregation state-specific antibodies may shed light on the KP-10-centric “fail-safe” mechanisms that partake in modulating α-syn proteostasis and expression levels. 

## 4. Materials and Methods

### 4.1. Kisspeptin Peptides

Human kisspeptin-10 (KP-10, H-YNWNSFGLRF-[NH2]; amino acids 112–121), obtained in lyophilized form (Tocris Bio-science, Bristol, UK; Cat. #: 2570), and kisspeptin-234 (KP-234, H-ANWNGFGWRF-[NH2]; a GPR54 antagonist), custom synthesized at a purity of ≥98% (Apical Scientific Sdn. Bhd., Selangor, Malaysia), were reconstituted in sterile water to yield a stock solution of 500 µM prior to subsequent dilution. 

### 4.2. α-Synuclein Plasmids

The pcDNA6 human wild-type (Insert size: 906 base pairs) and E46K mutant α-synuclein (α-syn, insert size: 1073 base pairs) plasmids were generously donated by Prof. Hilal Lashuel (Addgene, Water-town, MA, USA; plasmid nos. 107425 and 105730), whereas the enhanced green fluorescence protein (pEGFP-C1) control plasmid was attained from Clontech Laboratories (BD Biosciences, Palo Alto, CA, USA; plasmid no. 6084-1). Respective DNA sequences of PCR-amplified cloned inserts were next subjected to Sanger sequencing analyses for validation at Apical Scientific Sdn. Bhd. (Selangor, Malaysia). All plasmid constructs were purified with the Wizard^®^ Plus SV Minipreps DNA Purification System Kit (Promega, Madison, WI, USA; Cat. #: A1330) in due time.

### 4.3. Cell Culture

The SH-SY5Y neuroblastoma cell line procured from ATCC (Manassas, VA, USA; Cat. #: CRL-2266) was cultivated in 25 cm^2^ cell culture flasks at 37 °C under a moistened atmosphere of 5% CO^2^ in Dulbecco’s modified Eagle medium (DMEM) with high glucose and L-glutamine (Nacalai Tesque, Kyoto, Japan; Cat. #: 08469-35), supplemented with 10% fetal bovine serum (FBS) (Thermo Fisher Scientific, Waltham, MA, USA; Cat. #: 10100147) and 1% penicillin-streptomycin (MilliporeSigma, Burlington, MA, USA; Cat. #: P0781). The media were replenished every 2–3 days, and at a confluence of 70–90%, cells were harvested with Accutase (Nacalai Tesque, Kyoto, Japan; Cat. #: 12679-54), sub-cultivated into cell culture vessels, and replated for immunocytochemical and cell death assays. 

### 4.4. Differentiation into Cholinergic-like Neurons

SH-SY5Y-derived cholinergic-like neurons were generated using a differentiation methodology adapted from our previously documented protocol [22]. Concisely, SH-SY5Y cells were appropriately cultured in DMEM encompassing 0.5% FBS and 10 μM of all-trans retinoic acid (RA) (Sigma–Aldrich, St Louis, MO, USA; Cat. #: R2625) for 72 h to prompt neuronal differentiation. After three days in the presence of all-trans RA, the differentiation medium was replenished with DMEM containing 10% FBS for the transient transfection of plasmids. To that end, cells were never sub-cultured beyond passage 20 in order to circumvent any distinct phenotypic alteration in differentiated cholinergic cells.

### 4.5. Transient Transfections

The transient transfection of plasmid DNA in RA-differentiated cholinergic neurons was accomplished by employing Lipofectamine 3000 reagents (Thermo Fisher Scientific, Waltham, MA, USA; Cat. #: L3000001). Briefly, 2.5 μg of pEGFP-C1, 2.5 μg of pcDNA6 wild-type α-syn, or 2.5 μg of pcDNA6 E46K mutant α-syn plasmids were diluted in 125 μL of Opti-MEM serum-reduced medium (Thermo Fisher Scientific, Waltham, MA, USA; Cat. #: 31985070) and 5 μL of P3000 reagent, respectively. Succeeding a 5 min incubation step at room temperature (RT), 7.5 µL of Lipofectamine 3000 reagent was diluted in 125 μL of Opti-MEM serum-reduced medium, added into the diluted Opti-MEM-DNA solution, and incubated for an additional 15 min to assemble DNA-Lipofectamine 3000 lipoplexes. The complex concoctions were thereafter added dropwise into each well of a 6-well plate and re-incubated in a humidified 5% CO^2^ incubator for 2–4 days. The estimation of transfection efficiency in RA-differentiated cholinergic neurons was achieved by enumerating the amount of GFP-positive neurons under a Nikon Eclipse 90i fluorescent microscope (Nikon, Melville, NY, USA).

### 4.6. Quantification of Apoptosis by Annexin-V Labeling

Quantitative analysis of apoptotic profile in α-syn-overexpressing cholinergic cells was carried out using the Muse™ Annexin-V & Dead Cell Assay Kit (Luminex, Austin, TX, USA; Cat. #: MCH100105) in accordance with the manufacturer’s guidelines. Briefly, the assay utilizes fluorescently labeled annexin-V in combination with a dead cell marker, 7-Aminoactinomycin D (7-AAD), to detect the translocation of phosphatidylserine to the external membrane of apoptotic cells. SH-SY5Y cells were first seeded into 6-well plates (NEST Biotechnology, Wuxi, China; Cat. #: 703001) at a density of 2.5 × 10^5^ cells/well and cultured overnight. Following RA-induced differentiation and transient transfection of plasmids, cells were treated with KP-10 (0.1 µM) alone or in combination with KP-234 (0.1 to 10 µM) for 24 h in 5% CO^2^ at 37 °C. In this context, the selection of kisspeptin doses was based on previously published concentration-response curves and our very own preliminary observations [22]. Upon incubation, cells were harvested, gently rinsed with ice-cold PBS, collected by centrifugation at 1000 rpm for 5 min, and re-suspended in DMEM containing 1% FBS. The cell suspensions were then stained with 100 µL of Muse™ Annexin-V & Dead Cell Reagent and incubated for 20 min at RT in the dark. The events for live, early apoptotic, late apoptotic, and necrotic cells were finally analyzed by the Muse™ Cell Analyzer (Luminex, Austin, TX, USA). 

### 4.7. Mitochondrial Membrane Potential

Mitochondrial membrane depolarization in α-syn-overexpressing cholinergic cells was evaluated using the Muse™ MitoPotential Kit (Luminex, Austin, TX, USA; Cat. #: MCH100110) according to the manufacturer’s specifications. SH-SY5Y cells were initially plated into 6-well plates (NEST Biotechnology, Wuxi, China; Cat. #: 703001) at a density of 2.5 x 10^5^ cells/well and cultivated overnight. Upon RA-mediated differentiation and transient transfection of plasmids, cells were treated with KP-10 (0.1 µM) alone or in concert with KP-234 (0.1 to 10 µM) at 37 °C for 24 h in a 5% CO^2^ incubator. Following incubation, cells were harvested, centrifuged at 1000 rpm for 5 min, and re-suspended in 1X assay buffer. The Muse MitoPotential working solution was subsequently prepared by diluting the MitoPotential dye in 1X assay buffer (1:1000). Accordingly, 100 µL of cell suspension was added to 95 µL of MitoPotential working solution and incubated at 37 °C for 20 min. Eventually, 5 µL of 7-AAD reagent was methodically added, mixed thoroughly, and incubated for 5 min at RT. Lastly, changes in mitochondrial potential were determined by the Muse^®^ Cell Analyzer (Luminex, Austin, TX, USA).

### 4.8. Double Label Immunofluorescence

Cholinergic-like neurons overexpressing human wild-type or E46K mutant α-syn were firstly fixed with 4% paraformaldehyde in phosphate-buffered saline (PBS) (pH 7.4) for 20 min at RT. Prior to immunostaining, neurons were blocked and permeabilized with PBS containing 10% normal goat serum (NGS), 1% bovine serum albumin (BSA), and 0.3% Triton X-100 for 45 min. From there on, primary antibodies were applied sequentially and incubated overnight at 4 °C. The primary antibodies used were goat polyclonal anti-choline acetyltransferase (ChAT, 1:1000; MilliporeSigma, Burlington, MA, USA; Cat. #: AB144P) and rabbit monoclonal anti-α-syn (1:1000; Abcam, Cambridge, MA, USA; Cat. #: ab138501). After three washing steps with PBS, neurons were incubated with secondary antibodies, donkey anti-goat Alexa Fluor 594 (1:200; Thermo Fisher Scientific, Waltham, MA, USA; Cat. #: A-11058) and goat anti-rabbit Alexa Fluor 488 (1:200; Thermo Fisher Scientific, Waltham, MA, USA; Cat. #: A-11008) for 1 h at RT. Neuronal nuclei were next counterstained with 4′, 6-diamidino-2-phenylindole (DAPI, 1:1000; Thermo Fisher Scientific, Waltham, MA, USA; Cat. #: 62247) for 1 min. Immunostained cells were finally visualized at 20× magnification using an inverted Olympus IX81 microscope (Olympus America, Center Valley, PA, USA), while the semi-quantitative analyses of fluorescence intensities were successively calculated utilizing ImageJ plugins. 

### 4.9. Statistical Analysis

The experimental results were unveiled as mean ± SE from three independent biological experiments. Statistical analysis was appraised via one-way analysis of variance (ANOVA) prior to Tukey’s post hoc tests for all multiple assessments (IBM SPSS Statistics v24). A *p*-value of less than 0.05 was defined as a statistically significant difference. 

## Data Availability

The data presented in this study are available within the article. The raw data supporting the conclusions of this manuscript will be made available by the authors, without undue reservation, to any qualified researcher.
